# Announcement of the *Diagnostics* 2016 Junior Scientists Travel Award

**DOI:** 10.3390/diagnostics6020025

**Published:** 2016-06-15

**Authors:** Diagnostics Editorial Office

**Affiliations:** MDPI AG, Klybeckstrasse 64, CH-4057 Basel, Switzerland

With the goal of recognizing outstanding contributions to the field of medical diagnostics by early-career investigators, including assistant professors, postdoctoral students and PhD students, and assisting them in attending international conferences in 2016, early this year *Diagnostics* accepted nominations for the Junior Scientists Travel Award 2016. The nominations and applications were assessed by an Evaluation Committee consisting of senior scholars from the field.

We are excited to announce the winner, Dr. Jan Van den Stock, who will be supported with 800 Swiss Francs towards his travel expenses to attend international conferences in 2016.


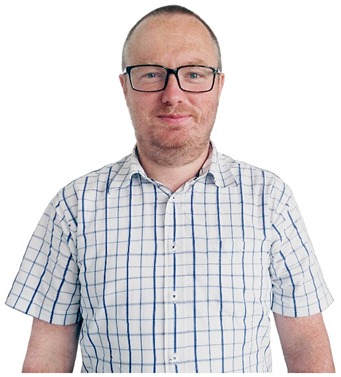


Dr. Jan Van den Stock is a clinical psychologist and post-doctoral FWO-fellow at KU Leuven, Belgium. His professional track record consists of a combination of research and clinical activities. His research focuses on the behavioral and neural correlates of socio-emotional processing in neurodegenerative disorders. Dr. Van den Stock combined his PhD-track with neuropsychological work in ambulant and residential neurology and psychiatry settings. In 2010, he obtained his PhD (Bodies and background: Contextual influences on perception of emotional body expressions). For his first postdoc term (FWO), he moved back from the Netherlands (Tilburg University) to Leuven and co-founded the laboratory headed by Prof. Dr. Mathieu Vandenbulcke to build on the work of his PhD. He investigated the behavioral and neural underpinnings of emotion and body perception in normal and clinical populations using psychophysical and functional and structural imaging [(f)MRI] techniques. Currently, Dr. Van den Stock is in the fifth year of his post-doc track. He has (co)authored 37 peer-reviewed articles and they are frequently cited (H-index = 15, according to Web of Science) and include recommendation by Faculty of 1000 (http://f1000.com/prime/13339967). Furthermore, he has organized research symposia with international speakers. He has taught five psychology and psychiatry courses at EHSAL Brussels (graduate school) and three neuropsychology and research courses at Tilburg University. He supervised graduate students for either their master’s thesis or research section of their clinical internship. He is co-promotor of two PhD-students. Regarding funding, he obtained grants from Tilburg University, FWO (post-doc fellowships and research grant), KU Leuven and Foundation for Alzheimer Research (SAO-FRA). Furthermore, he has been solicited as a reviewer for leading journals like *PNAS*, *Biological Psychiatry*, *Current Biology*, *eLife*, *NeuroImage*… This year, he received a special reviewer recommendation from the editorial board of PLoS ONE.

We wish to congratulate Dr. Jan Van den Stock for his accomplishments.

